# Broccoli Sprout Extract Suppresses Particulate-Matter-Induced Matrix-Metalloproteinase (MMP)-1 and Cyclooxygenase (COX)-2 Expression in Human Keratinocytes by Direct Targeting of p38 MAP Kinase

**DOI:** 10.3390/nu16234156

**Published:** 2024-11-30

**Authors:** Jaehyeok Yun, Jong-Eun Kim

**Affiliations:** Department of Food Science and Technology, Korea National University of Transportation, Jeungpyeong 27909, Republic of Korea; qweasd126@naver.com

**Keywords:** particulate matter (PM), skin, broccoli sprout extract (BSE), p38

## Abstract

Background/Objectives: Particulate matter (PM) is an environmental pollutant that negatively affects human health, particularly skin health. In this study, we investigated the inhibitory effects of broccoli sprout extract (BSE) on PM-induced skin aging and inflammation in human keratinocytes. Methods: HaCaT keratinocytes were pretreated with BSE before exposure to PM. Cell viability was assessed using the MTT assay. The expression of skin aging and inflammation markers (MMP-1, COX-2, IL-6) was measured using Western blot, ELISA, and qRT-PCR. Reactive oxygen species levels were determined using the DCF-DA assay. Kinase assays and pull-down assays were conducted to investigate the interaction between BSE and p38α MAPK. Results: Our findings demonstrate that BSE effectively suppressed the expression of MMP-1, COX-2, and IL-6—critical skin aging and inflammation markers—by inhibiting p38 MAPK activity. BSE binds directly to p38α without competing with ATP, thereby selectively inhibiting its activity and downstream signaling pathways, including MSK1/2, AP-1, and NF-κB. Conclusions: These results suggest that BSE is a potential functional ingredient in skincare products to mitigate PM-induced skin damage.

## 1. Introduction

The skin is directly exposed to the external environment and acts as a physical barrier that protects the human body from physical, chemical, and biological threats to the atmosphere [1]. Ultraviolet radiation has been predominantly implicated as a factor affecting the skin; recent studies have shown that particulate matter (PM) also plays a significant role [2,3]. PM is a term encompassing dust particles that are not visible to the naked eye and are classified based on size into PM10 (10 μm or less) and PM2.5 (2.5 μm or less) [4]. PM consists of nitrates, sulfates, elements, elemental carbon, organic compounds, biological compounds, and metals. It is primarily generated by industrial activities in factories and power plants [5]. Due to their extremely small size, PM can easily penetrate the human body. PM entering the body can adversely affect the respiratory and cardiovascular systems and pose a threat to human health [6]. Similarly, PM exerts various effects on the skin. PM penetration weakens the structure and function of the skin, inducing skin barrier breakdown, cell death, inflammation, and various disorders, thereby threatening skin health [7,8,9,10,11].

With the negative impacts of PM becoming evident, global efforts to reduce its generation are underway; however, significant time and collective efforts are required. Hence, there is an increasing focus on functional foods that can effectively mitigate the effects of PM at the individual level [12]. Plants have long been regarded as natural skin care sources and for maintaining health. Plants capable of reducing cellular oxidative stress can provide valuable strategies to inhibit PM-induced skin inflammation and aging caused by PM [13]. Broccoli (*Brassica oleracea* var. italica), a member of the Brassicaceae family, originated in the eastern Mediterranean and is now widely cultivated and consumed worldwide [14,15]. Broccoli contains numerous bioactive compounds, including glucosinolates, polyphenols, carotenoids, minerals, and vitamins [16,17]. Broccoli sprouts contain higher amounts of bioactive compounds than fully grown broccoli. Hence, research investigating broccoli sprouts is being actively conducted [18]. Broccoli sprouts possess potent antioxidant activity due to various bioactive components that contribute to their ability to prevent cell death and inflammation [19,20,21]. Additionally, it has been revealed to effectively prevent cancer development with various beneficial effects [22,23,24,25,26].

Particulate matter upregulates the expression of matrix-metalloproteinase (MMP)-1 and cyclooxygenase (COX)-2 in the skin [27,28]. MMP-1 has been reported to impact skin aging significantly, and its expression is primarily increased by UV exposure [29,30]. MMP-1 plays a crucial role in the degradation of the extracellular matrix (ECM), including collagen [31]. It promotes the breakdown of collagen types I and III, negatively affecting ECM integrity [32]. Collagen constitutes 90% of the dermis and plays a crucial role in skin structure and tensile strength [33]. However, its degradation leads to the loss of skin elasticity and wrinkle formation, ultimately resulting in skin aging [34,35]. Inflammation is a physiological process that responds to tissue damage caused by microbes, pathogens, chemical irritants, or wounds and is a protective mechanism that maintains skin homeostasis from external stimuli [36,37]. COX-2 is expressed in response to inflammatory reactions and acts as an enzyme that catalyzes the conversion of arachidonic acid to prostaglandin E_2_ (PGE_2_), which can induce inflammation and skin cancer [38,39]. Excessive COX-2 expression induces chronic inflammation that adversely affects skin health [40]. The expression of MMP-1 and COX-2 is primarily regulated by the MAPK signaling pathway that plays an essential role in many biological processes, including inflammation, cell death, proliferation, and differentiation [31,41]. In previous studies, exposure increases the activities of extracellular signal-regulated kinase (ERK) 1/2, c-Jun N-terminal kinase (JNK) 1/2, and p38 MAPK in human keratinocytes [8]. The MAPK signaling pathway is a significant mediator of MMP-1 and COX-2 expression induced by PM, and control of the MAPK signaling pathway can be an excellent strategy for suppressing skin aging and inflammation [42].

MSK1/2 (Mitogen- and Stress-Activated Protein Kinases 1 and 2) are downstream targets of the p38 MAP kinase and play crucial roles in mediating inflammatory responses [43]. When p38 is activated by environmental stressors, including particulate matter, it phosphorylates and activates MSK1/2. This activation promotes the expression of pro-inflammatory mediators, such as cytokines and matrix metalloproteinases, which contribute to skin inflammation and damage [44]. Therefore, controlling the p38-MSK1/2 signaling axis represents a potential strategy to mitigate inflammation and other inflammatory conditions triggered by external environmental factors [45]. In this study, we explore how targeted inhibition of this pathway may reduce the inflammatory impact of particulate matter on skin health.

Although broccoli sprouts have been demonstrated to exhibit various beneficial effects, their effects on skin aging and PM-induced inflammation remain unclear. Therefore, in this study, we utilized human keratinocyte cells (HaCaT cells) in vitro to investigate the effects of broccoli sprouts on MMP-1 and COX-2 expression induced by PM and to examine the molecular mechanisms involved.

## 2. Results

### 2.1. Effects of Broccoli Sprout Extract (BSE) on PM-Induced Skin Aging in HaCaT Cells

**Figure 1:**
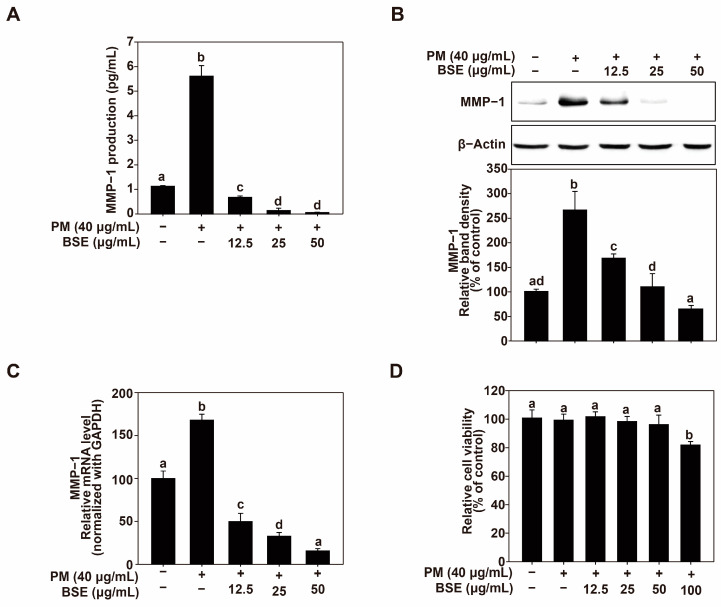
Effects of BSE on PM-induced MMP-1 production and expression. (**A**) Influence of BSE on MMP-1 production in HaCaT cells. HaCaT cells were pretreated with varying concentrations of BSE for 1 h prior to PM exposure (40 μg/mL). After 24 h, culture media was collected, and MMP-1 levels were quantified using ELISA kits following the protocol in the Materials and Methods section. (**B**) Influence of BSE on MMP-1 expression in HaCaT cells. Cells were pretreated with specified concentrations of BSE for 1 h before PM exposure (40 μg/mL). Following a 24 h incubation, cells were lysed as outlined in Materials and Methods, and MMP-1 protein levels were assessed via Western blotting. β-Actin was used as a loading control. Images were analyzed using the FUSION Solo S system (VILBER Lourmat, Paris, France). (**C**) Effect of BSE on MMP-1 mRNA levels in HaCaT cells. Quantitative real-time PCR was used to assess MMP-1 mRNA expression following 1 h pretreatment with BSE at specified concentrations and 6 h PM exposure (40 μg/mL). Cells were lysed according to the Materials and Methods. Data (*n* = 3) are presented as mean ± SD. (**D**) Impact of BSE on HaCaT cell viability. Cell viability was evaluated using the MTT assay 24 h after pretreatment with BSE at indicated concentrations and PM exposure (40 μg/mL). Data (*n* = 5) are shown as mean ± SD. Different letters (a–d) on the graph indicate statistically significant differences (*p* < 0.05) determined by one-way ANOVA, with Duncan’s Multiple Range test as a post hoc test.

MMP-1 is a collagenase that decomposes collagen types I and III, directly influencing skin elasticity and wrinkle formation [46]. Therefore, MMP-1 inhibition can prevent skin aging by protecting the skin against collagen. Based on this, we investigated the effects of BSE on PM-induced MMP-1 expression. BSE treatment effectively inhibited PM-induced MMP-1 production in HaCaT cells (Figure 1A) without affecting cell viability (Figure 1D). To identify the mechanism underlying MMP-1 inhibition, the intracellular MMP-1 expression was investigated. BSE treatment downregulated MMP-1 expression in a dose-dependent manner (Figure 1B), and BSE also inhibited mRNA expression of MMP-1 in a dose-dependent manner. These results indicated that BSE effectively suppressed PM-induced MMP-1 expression in HaCaT cells.

### 2.2. BSE Attenuates PM-Induced Inflammation

**Figure 2:**
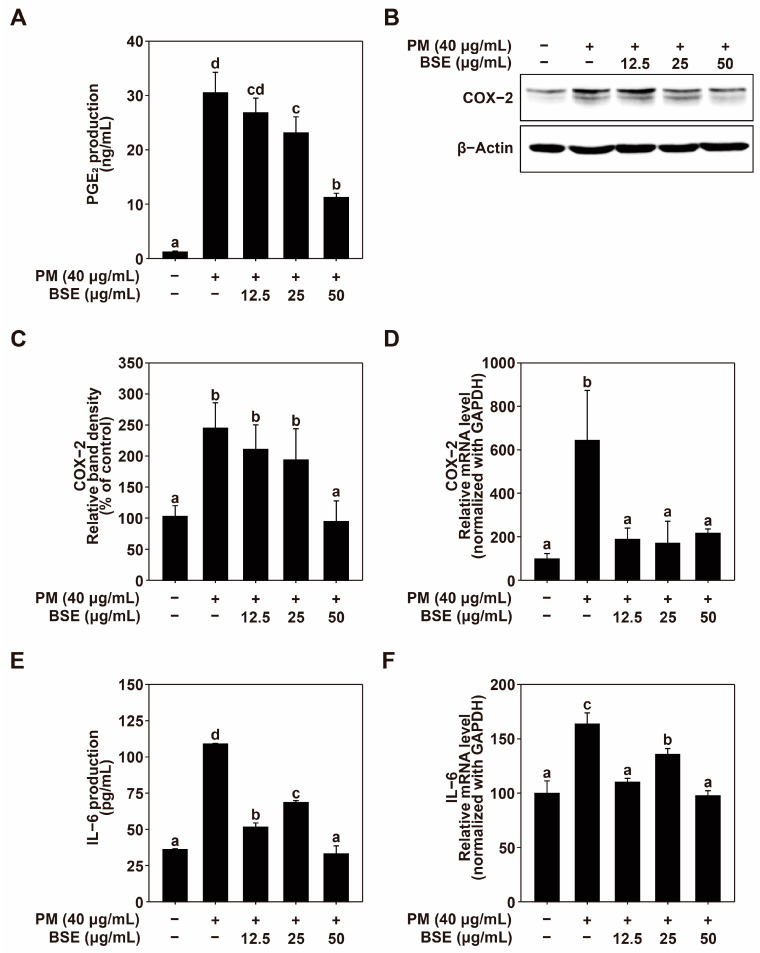
Effects of BSE on PM-induced COX-2 and IL-6 production and expression. (**A**) Influence of BSE on PGE2 production in HaCaT cells. HaCaT cells were pretreated with various concentrations of BSE for 1 h before PM exposure (40 μg/mL). Following 24 h, culture media was collected, and PGE2 production was measured using enzyme immunoassay kits as detailed in the Materials and Methods. (**B**,**C**) Influence of BSE on COX-2 expression in HaCaT cells. Cells were pretreated with the indicated concentrations of BSE for 1 h prior to PM exposure (40 μg/mL). After 24 h, cells were lysed as per the protocol, and COX-2 protein levels were analyzed by Western blotting. β-Actin was used as a loading control. The FUSION Solo S imaging system (VILBER Lourmat, Paris, France) was used for measurements. (**D**) Effect of BSE on COX-2 mRNA levels in HaCaT cells. Quantitative real-time PCR was employed to assess COX-2 mRNA levels following 1 h BSE pretreatment and 6 h PM exposure (40 μg/mL). Cells were lysed as described in the Materials and Methods. (**E**) Effect of BSE on IL-6 production in HaCaT cells. Cells were pretreated with BSE for 1 h before exposure to PM (40 μg/mL), and after 24 h, IL-6 levels in the culture media were quantified using ELISA kits as per the protocol. (**F**) Influence of BSE on IL-6 mRNA expression in HaCaT cells. Quantitative real-time PCR was used to assess IL-6 mRNA levels after 1 h BSE pretreatment and 6 h PM exposure (40 μg/mL). Data (*n* = 3) are shown as mean ± SD. Means with different letters (a–d) indicate significant differences (*p* < 0.05), determined by one-way ANOVA, with Duncan’s Multiple Range test as the post hoc test.

COX-2 is directly involved in the production of PGE_2_ and is an enzyme that is primarily expressed during inflammatory reactions. It can adversely affect skin health by inducing chronic inflammation [40]. Cytokines are key mediators that regulate the immune system and play essential roles in various physiological processes. Among them, IL-6 is considered to be an inflammatory cytokine and a crucial biomarker for inflammation control due to its involvement in most inflammatory responses. PM upregulates the expression of IL-6 in the skin, and the IL-6 thus produced can subsequently induce other inflammatory reactions [47]. Overall, the inhibition of the expression of COX-2 and IL-6 induced by PM can reduce excessive inflammatory reactions. Therefore, we investigated the effect of BSE on the PM-induced expression of COX-2 and IL-6. BSE treatment downregulated PM-induced PGE_2_ production in HaCaT cells in a dose-dependent manner (Figure 2A). To identify the inhibition mechanism of PGE_2_, COX-2 expression was investigated. Treatment with BSE at 50 μg/mL effectively inhibited PM-induced COX-2 expression (Figure 2B,C), and BSE treatment effectively suppressed COX-2 mRNA expression. Moreover, SBE treatment significantly attenuated PM-induced IL-6 production and mRNA expression (Figure 2E,F). These results indicated that SBE could effectively inhibit PM-induced inflammation in HaCaT cells.

### 2.3. BSE Inhibits PM-Induced AP-1 and NF-κB Activity

**Figure 3:**
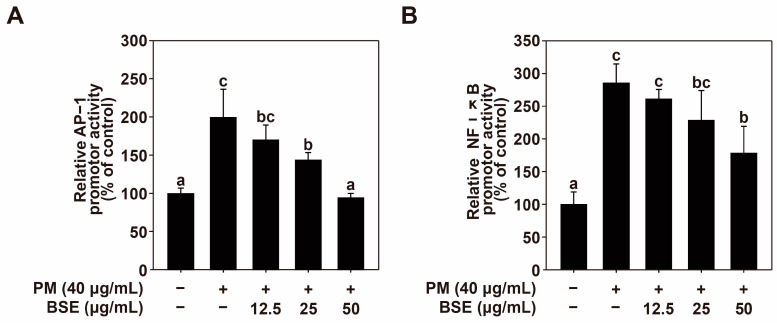
Effect of BSE on PM-induced AP-1 and NF-κB activity in transactivated cells. The transactivation of (**A**) AP-1 and (**B**) NF-κB was assessed using a GFP reporter gene assay. HaCaT cells transduced with AP-1 and NF-κB were pretreated with BSE at specified concentrations for 1 h prior to PM exposure (40 μg/mL). After 24 h, the transactivation was measured at an excitation wavelength of 469 nm and an emission wavelength of 525 nm (GFP). Data (*n* = 3) are presented as the mean ± SD. The means labeled with different letters (a–c) on the graph indicate significant differences (*p* < 0.05) according to one-way ANOVA, with Duncan’s Multiple Range Test used for post hoc analysis.

It has been reported that PM-induced MMP-1 transcription is regulated by AP-1 [48], while COX-2 and IL-6 are regulated by NF-κB [28,49]. Based on these findings, we investigated the activities of AP-1 and NF-κB following BSE treatment. BSE treatment decreased the PM-induced AP-1 (Figure 3A) and NF-κB activities (Figure 3B). These results suggest that BSE inhibits PM-induced MMP-1, COX-2, and IL-6 transcription by suppressing AP-1 and NF-κB activity.

### 2.4. BSE Inhibits MSK1/2 Phosphorylation in HaCaT Cells

**Figure 4:**
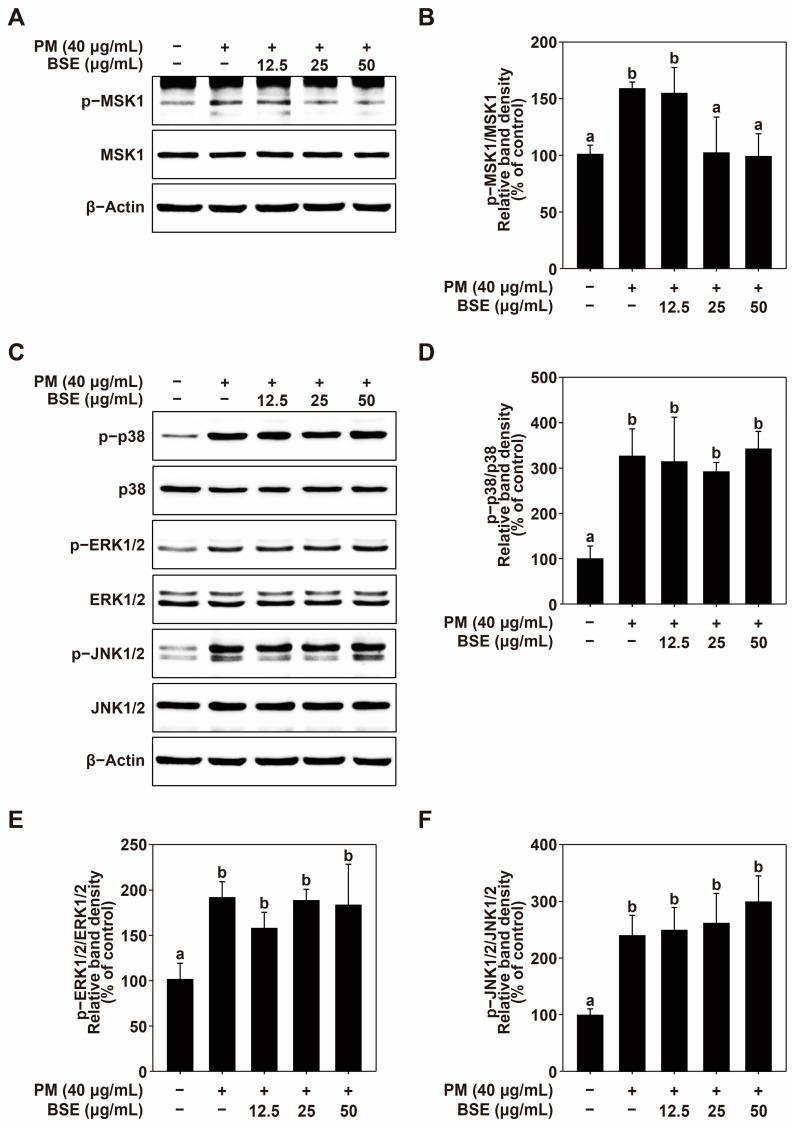
Effect of SBE on PM-induced MSK1 phosphorylation in HaCaT cells. Effects of BSE on PM-induced phosphorylation of (**A**,**B**) MSK1/2, (**C**,**D**) p38, (**C**,**E**) ERK1/2, and (**C**,**F**) JNK1/2. HaCaT cells were pretreated with BSE at the indicated concentrations for 1 h before exposure to PM (40 μg/mL). After 2 h, the HaCaT cells were lysed as described in the Materials and Methods. Phosphorylated and total protein levels were analyzed by Western blotting. Images were measured with FUSION Solo S (VILVER Lourmat, Paris, France). Data (*n* = 3) are presented as the mean ± SD. The means with letters (a,b) on the graph indicate a significant difference (*p* < 0.05) according to one-way ANOVA, and Duncan’s Multiple Range test was used as a post hoc test.

The MAPK signaling pathway is well known to regulate the activity of AP-1 and NF-κB [31,41]. Therefore, the effect of BSE on PM-induced phosphorylation of MAP kinases was investigated. BSE treatment effectively inhibited MSK1/2 phosphorylation in HaCaT cells (Figure 4A,B). However, BSE did not suppress the phosphorylation of p38 and ERK1/2 (Figure 4C–E) that are upstream of MSK1/2, nor did it inhibit the phosphorylation of JNK1/2 (Figure 4C,F).

### 2.5. BSE Inhibits p38 MAPK Activity Through Direct Binding

**Figure 5:**
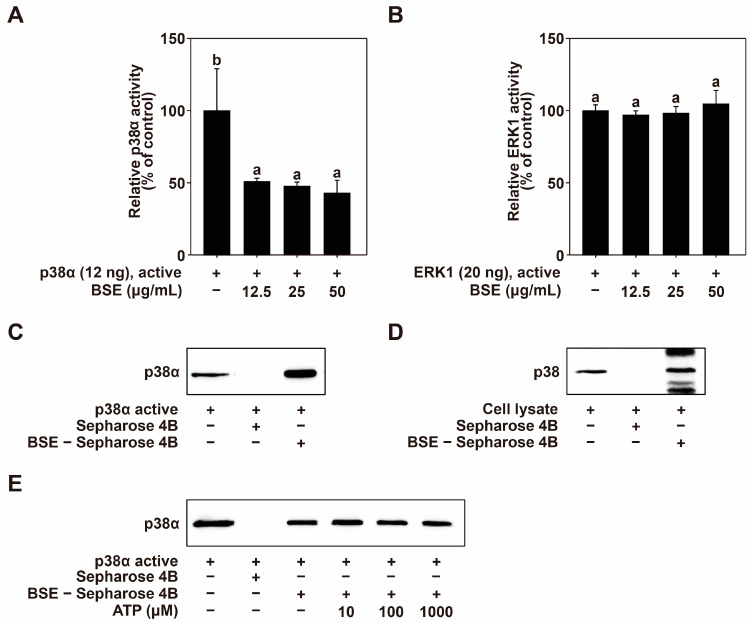
Effects of BSE on p38 MAPK activity via direct binding. (**A**) BSE inhibits p38α activity. (**B**) BSE does not inhibit ERK1 activity. Kinase activity was measured as outlined in the Materials and Methods section. (**C**) Interaction between BSE and active p38α. Binding of BSE to p38α was confirmed by Western blot analysis using an anti-p38α antibody: Lane 1 (input control) contains 10 ng of p38α, serving as a protein standard. Lane 2 (control) contains Sepharose 4B beads. Lane 3 shows the p38α protein pulled down by BSE conjugated to Sepharose 4B beads. (**D**) Binding of BSE to p38 in HaCaT cell lysate. Western blotting, using an anti-p38 antibody, confirmed the binding: Lane 1 (input control) contains 0.5 µg of HaCaT cell lysate as a protein standard. Lane 2 (control) contains Sepharose 4B beads. Lane 3 indicates that p38 was pulled down by BSE–Sepharose 4B beads. (**E**) BSE does not compete with ATP for binding to p38α. Lane 1 (input control) includes 10 ng of p38α, used as a protein standard. Lane 2 (control) contains Sepharose 4B beads. Lane 3 shows p38α pulled down by BSE–Sepharose 4B beads, while Lanes 4-6 demonstrate active p38α pulled down by BSE–Sepharose 4B beads in the presence of ATP at concentrations of 10, 100, and 1000 µM. The means with letters (a,b) on the graph indicate a significant difference (*p* < 0.05) according to one-way ANOVA, and Duncan’s Multiple Range test was used as a post hoc test.

p38 and ERK1/2 play important roles in MSK1/2 activation. Therefore, the regulation of p38 and ERK1/2 activities affects MSK1/2 phosphorylation [50]. BSE inhibits the phosphorylation of MSK1/2 but does not suppress the phosphorylation of p38 or ERK1/2. Therefore, the effect of BSE on the activity of p38α and ERK1 was investigated. Using a kinase assay, we demonstrated that BSE effectively inhibited the activation of p38α (Figure 5A) but did not suppress the activation of ERK1/2 (Figure 5B). These findings suggest that the regulation of MSK1/2 phosphorylation occurred due to the regulation of p38α activation rather than that of ERK1. To identify how BSE modulates p38α activity, we investigated whether BSE binds directly to p38α. To determine if BSE binds directly to p38α, we performed pull-down assays. The pull-down assay results suggest that BSE physically interacts with the active p38α protein (Figure 5C) but not with unconjugated Sepharose 4B beads. Additionally, the direct binding of BSE to p38 was confirmed in HaCaT cell lysate (Figure 5D). Next, we performed ATP competition assays to examine the mode of BSE binding to p38α. ATP non-competed with BSE for p38α binding (Figure 5E). These results indicate that BSE binds to p38α at allosteric sites.

### 2.6. Antioxidant Effects and Contents of Total Phenolics in BSE

**Figure 6:**
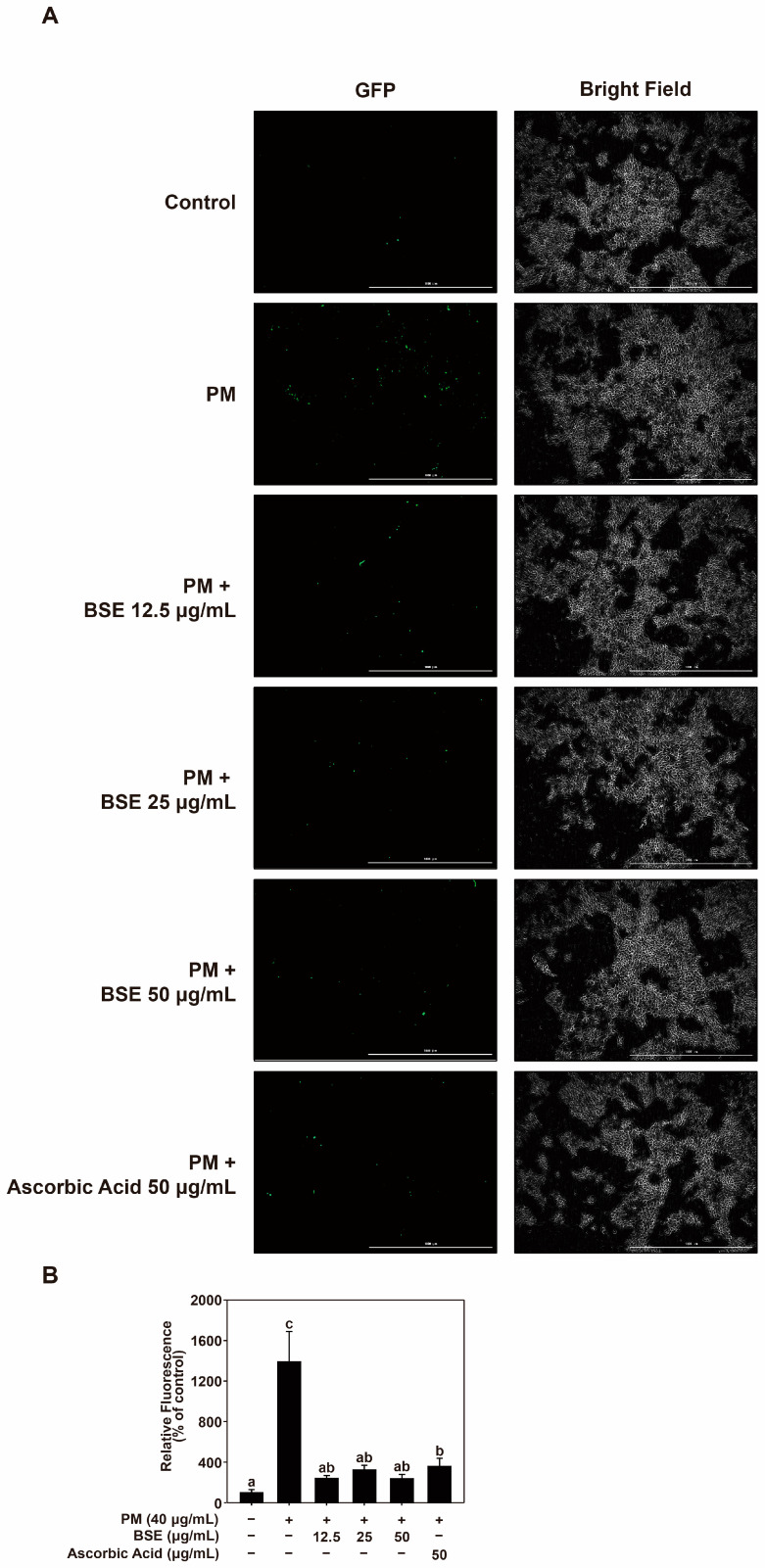
Effect of BSE on PM-induced ROS production. (**A**,**B**) Images and quantification of PM-induced ROS reduction by BSE were determined using a DCF-DA fluorescence assay. Data (*n* = 3) are expressed as the mean ± SD. Different letters (a–c) on the graph indicate significant differences (*p* < 0.05), as determined by one-way ANOVA followed by Duncan’s Multiple Range Test for post hoc analysis.

PM also increases oxidative stress, which is characterized by increased ROS levels in the skin [49,51]. ROS activate several signaling pathways and play a pivotal role in activating the MAPK signaling pathway [52]. Therefore, antioxidants that suppress ROS production are useful for alleviating the effects of PM. Based on this, we investigated the antioxidant and inhibitory effects of BSE on PM-induced ROS production. To confirm the antioxidant properties of BSE, its antioxidant capacity was assessed using ABTS and DPPH assays. It was compared to vitamin C and marked as a vitamin C equivalent. Additionally, the phenol content with excellent antioxidant capacity was investigated using the TPC assay, compared to tannic acid, and marked as tannic acid equivalents (Table 1). BSE effectively suppressed PM-induced ROS production (Figure 6A,B). Our results suggest that BSE effectively reduces ROS levels and may provide relief from skin aging and inflammation associated with PM exposure.

## 3. Discussion

With the growing awareness of the dangers posed by PM, concerns regarding skin health due to PM pollution are increasing [12]. Naturally sourced plants may serve as a promising strategy against skin contamination caused by PM [13]. Broccoli sprouts, known for their antioxidant, anti-inflammatory, and anticancer properties, have been reported to exhibit various physiological activities that are potentially beneficial to human health [19,21,53]. Therefore, our objective was to investigate the efficacy of broccoli sprouts against skin aging and inflammation induced by PM and to elucidate the underlying mechanisms.

BSE are rich in bioactive compounds with significant health benefits. They contain high levels of glucosinolates and isothiocyanates, particularly sulforaphane. Sulforaphane is known to activate antioxidant and detoxification enzymes, which may contribute to cancer prevention [54]. In addition to sulforaphane, BSE are abundant in antioxidant substances such as phenolic compounds, flavonoids, and sinapic acid derivatives. These compounds help reduce oxidative stress and inflammation, potentially playing a role in the prevention of chronic diseases [55]. Sulforaphane-related derivatives, including 5-methylsulfinylpentyl nitrile and 4-methylsulfinylbutyl nitrile, have demonstrated activity in inhibiting *Helicobacter pylori*, a bacterium associated with gastric ulcers and cancer [56]. Moreover, BSE contain complex anthocyanins and other polyphenolic components that provide antioxidant and anti-inflammatory effects [57]. These compounds contribute to the overall health benefits associated with BSE consumption.

We established that BSE effectively inhibited PM-induced MMP-1, COX-2, and IL-6 expression, thus demonstrating its anti-aging and anti-inflammatory effects. Investigation of the underlying mechanism indicated that this effect could be attributed to the inhibition of p38 kinase activation. p38 MAPK influences various intracellular responses, playing an established role in inflammation, cell cycle regulation, apoptosis, embryonic development, cellular differentiation, aging, and tumor formation [58]. Previous studies have revealed that PM-induced phosphorylation of p38 increases the mRNA and protein expression of MMP-1, COX-2, and IL-6 in HaCaT cells and HDF [59]. Based on this evidence, we first examined the p38 isoforms. There are four isoforms of p38 (α, β, γ, and δ), with p38α being the most abundantly expressed isoform in most cell types. p38β is expressed at much lower levels compared to that of p38α and is considered to possess similar functions to those of p38α. Additionally, the expression patterns of p38γ and p38δ are more limited [60]. Therefore, in this study, research examining p38 was conducted using p38α due to its widespread expression. Through kinase assay results, it was confirmed that BSE effectively inhibits the activity of p38α. Furthermore, the pull-down assay results revealed that BSE directly binds to recombinant p38α and p38 from HaCaT cell lysate and exhibits non-competitive binding with ATP. Inhibition of p38 activity led to the suppression of phosphorylation of downstream targets, MSK1, and subsequent inhibition of AP-1 and NF-kB activity [43,61,62]. Ultimately, we concluded that BSE suppressed the expression of MMP-1, COX-2, and IL-6 by inhibiting p38 activity. The structural biology of p38 MAPK provides a crucial understanding of how inhibitors such as BSE exert their effects. The p38 MAPK protein consists of a conserved kinase domain characterized by a bilobed structure typical of protein kinases. The active site situated between these lobes is where ATP binding and substrate phosphorylation occur. Traditional ATP-competitive inhibitors bind directly to this active site, often leading to broad-spectrum inhibition that can result in off-target effects and toxicity.

However, BSE, similar to certain other selective inhibitors, operates through a non-ATP competitive mechanism by binding to an allosteric site on p38α. Structural studies have revealed that this allosteric site is distinct from the ATP-binding pocket, typically located near regions such as the docking site for ERK and FXF motifs (DEF) and the common docking (CD) domains that are crucial for substrate and regulatory protein interactions. The binding of BSE to this allosteric site induces conformational changes in the kinase that prevent its activation without directly blocking ATP binding. This mechanism is advantageous, as it allows for the selective inhibition of p38α, potentially preserving the functions of other p38 isoforms and reducing the likelihood of adverse effects [63].

In the context of p38 MAPK, structural biology studies have highlighted the ability of allosteric inhibitors to stabilize the inactive conformations of kinases, thereby reducing their activity. This stabilization is particularly relevant in the context of inflammatory diseases in which excessive p38 MAPK activity contributes to pathogenesis [58]. By targeting specific conformations of p38α, BSE can effectively inhibit its activity, leading to reduced phosphorylation of downstream targets like MSK1/2 and subsequent suppression of pro-inflammatory pathways [62]. This structural approach to inhibition underscores the therapeutic potential of non-ATP competitive inhibitors such as BSE, offering a targeted means of modulating kinase activity with potentially fewer side effects than those caused by traditional inhibitors. Future structural studies should focus on elucidating the precise binding interactions of BSE with p38α and determine how these interactions translate to its inhibitory effects at the molecular level. This addition to the discussion provides a deeper understanding of how BSE interacts with p38 MAPK at the structural level, emphasizing its unique mechanism of action and potential advantages over conventional ATP-competitive inhibitors. BSE is a complex extract containing numerous compounds, which may act through diverse mechanisms. However, the experimental results demonstrate direct inhibition of p38 by BSE. Therefore, at the tested concentrations, this mechanism can be considered a plausible and appropriate explanation for its observed effects.

It has been reported that the effects of PM on the skin are primarily due to the generation of reactive oxygen species (ROS) [49]. The generated ROS affect various signaling pathways, particularly by inducing phosphorylation and subsequent activation of the MAPK signaling pathway [52]. In this study, BSE suppressed PM-induced ROS to the level of the untreated group and exhibited a superior effect compared to that of ascorbic acid, which was used as a positive control. However, the suppression of ROS by BSE did not decrease the phosphorylation of the MAPK signaling pathway. These results suggest that the physiological response of cells to PM is not linear but results from complex interactions involving ROS [64]. PM generates ROS not only through mitochondrial damage but also by directly binding to Toll-like receptor (TLR) 5 that mediates phosphorylation of the MAPK signaling pathway [65,66]. Additionally, benzo[a]pyrene, identified as a component of PM, induces the activation of the AhR signaling pathway that, in turn, mediates the phosphorylation of the MAPK signaling pathway [67,68]. 

### Conclusion

PM directly affects various signaling pathways, resulting in complex interactions. Collectively, the effect of BSE does not appear to be merely due to ROS suppression. Instead, its ROS-inhibitory effect appears to be mitigated by complex cellular signaling pathways. This indicates that further investigation of the effects of PM is necessary.

## 4. Materials and Methods

### 4.1. Chemicals and Materials

High-glucose Dulbecco’s Modified Eagle’s Medium (DMEM), penicillin-streptomycin, and trypsin-EDTA were sourced from Welgene (Gyeongsan, Republic of Korea). Ascorbic acid was acquired from LPS Solution (Daejeon, Republic of Korea). Fetal bovine serum (FBS) was obtained from Atlas Biologicals (Fort Collins, CO, USA). Antibodies against total p38 MAPK, phosphorylated ERK1/2 (Thr202/Tyr204), total JNK1/2, total MSK1, and β-actin were from Santa Cruz Biotechnology (Santa Cruz, CA, USA). The MMP-1 antibody was purchased from R&D Systems Inc. (Minneapolis, MN, USA). Other antibodies came from Cell Signaling Technology (Danvers, MA, USA). MTT powder was obtained from USB Corporation (Cleveland, OH, USA). DCF-DA was from Sigma-Aldrich (St. Louis, MO, USA). Lentiviral vectors, pGF-AP1-mCMV-EF1-Puro, and pGF-NF-κB-mCMV-EF1-Puro, along with packaging vectors pMD2.G and psPAX2, were sourced from Addgene Inc. (Cambridge, MA, USA). The ECL Prime Western Blotting Detection Reagent was from Amersham (Little Chalfont, UK). Recombinant active p38α and ERK1 were obtained from SignalChem Biotech Inc. (Richmond, CA, USA).

### 4.2. Broccoli Sprout Extract (BSE) Preparation

Broccoli sprout extract (BSE) was provided by the Korea Ginseng Farming Association. The extraction involved washing broccoli sprouts and adding distilled water (ddH_2_O). The soluble compounds were extracted by heating at 85 °C for 24 h. The extract was subsequently freeze-dried using a freeze dryer (FD8508, Ilshin Biobased, Jeonju, Republic of Korea), stored in powdered form at −80 °C, and used as needed.

### 4.3. Antioxidant Properties and Phenolic Content of BSE

The antioxidant properties, including ABTS and DPPH radical scavenging activities, and the total phenolic content were determined following Lee’s method [43]. For the ABTS assay, 245 mM potassium persulfate was combined with 7 mM ABTS to generate ABTS•^+^ radicals. After 12 h, 50 μL of the mixture was added to 100 μL ddH_2_O and incubated in darkness for 5 min. The absorbance at 734 nm was measured with a microplate reader (Biotek, Winooski, VT, USA), with the optical density adjusted between 0.7 and 0.9 at 734 nm. For the DPPH assay, a 0.2 mM solution of DPPH in 99% ethanol was incubated in the dark for 10 min, and absorbance was measured at 517 nm, with an optical density range of 0.5 to 0.7. The total phenolic content was evaluated by adding Folin-Ciocalteu reagent, incubating for 5 min in the dark, followed by the addition of 7% Na_2_CO_3_ solution and a further 30 min incubation, after which the absorbance was measured at 750 nm.

### 4.4. Cell Culture

HaCaT keratinocytes obtained from CLS Cell Lines Services GmbH (Heidelberg, Germany) were cultured at 37 °C in a humidified 5% CO_2_ environment in DMEM supplemented with 10% FBS and 1% antibiotics. Cells were subcultured when reaching 80–90% confluence to maintain viability.

### 4.5. Particulate Matter (PM) Preparation

Particulate matter (PM) was sourced from Sigma-Aldrich (St. Louis, MO, USA). PM was prepared at 25 mg/mL in DMSO and sonicated for 40 min to prevent agglomeration. For experiments, the PM stock was diluted in serum-free DMEM.

### 4.6. Cell Viability Assay

Cell viability was assessed using the MTT assay. HaCaT cells were seeded in 96-well plates and incubated with serum-free DMEM for 24 h. Cells were pretreated with BSE for 1 h before exposure to PM. After 24 h, MTT solution (0.45 mg/mL) was added, and cells were incubated at 37 °C and 5% CO_2_ for 3 h. The formazan crystals were dissolved in 100 μL of DMSO, and absorbance at 570 nm was measured using a microplate reader (Biotek, VT, USA).

### 4.7. Western Blot Analysis

HaCaT cells cultured in 6-well plates were incubated with serum-free DMEM for 24 h, pretreated with BSE for 1 h, then treated with PM. Cell lysates were prepared in cell lysis buffer [20 mM Tris-HCl (pH 7.5), 150 mM NaCl, 1 mM Na_2_EDTA, 1 mM EGTA, 1% Triton X-100, 2.5 mM sodium pyrophosphate, 1 mM β-glycerophosphate, 1 mM Na_3_VO_4_, 1 μg/mL leupeptin] (Cell Signaling Technology, Danvers, MA, USA), collected on ice, and centrifuged at 12,970 rpm for 10 min. Protein concentrations were measured using a Bio-Rad protein assay kit (Hercules, CA, USA). Proteins were resolved on 6%, 8%, or 10% SDS-PAGE gels and transferred to PVDF membranes (0.45 μm pore size; Amersham, UK). Membranes were blocked with 5% BSA or milk for 1 h, incubated with specific primary antibodies overnight at 4 °C, and then treated with HRP-conjugated secondary antibodies (GenDepot, Katy, TX, USA). Protein visualization was performed using ECL Prime, and protein bands were quantified with an image analysis system (Vilber Lourmat, Marne-la-Vallée, France).

### 4.8. ELISA for MMP-1 and IL-6

MMP-1 and IL-6 levels in the culture medium were measured using ELISA kits from R&D Systems Inc. (MN, USA) following the manufacturer’s instructions.

### 4.9. PGE_2_ Measurement

PGE_2_ levels in the medium were quantified using a PGE_2_ enzyme immunoassay kit from Cayman Chemical Company (Ann Arbor, MI, USA) according to the manufacturer’s protocol.

### 4.10. Quantitative Real-Time PCR (qRT-PCR)

Total RNA was extracted from HaCaT cells using RNAiso Plus (Takara Bio Inc., Shiga, Japan), and RNA concentration and purity were assessed with a Take3 plate (Biotek, VT, USA). cDNA synthesis was performed using a PrimeScript™ 1st Strand cDNA Synthesis Kit (Takara Bio Inc., Japan). qRT-PCR was conducted with TB Green^®^ Premix Ex Taq™ using specific primers for COX-2, MMP-1, IL-6, and GAPDH. Amplification was performed in triplicate, with GAPDH as an internal control.

### 4.11. Measurement of ROS

Reactive oxygen species (ROS) levels were measured with the DCF-DA assay. HaCaT cells cultured in 96-well black plates were treated with DCF-DA (100 μM) for 30 min, rinsed with HBSS, and then treated with PM and BSE. DCF fluorescence was detected using a Cell Imaging Multi-Mode Reader (Biotek, VT, USA) at an excitation wavelength of 469 nm and an emission wavelength of 525 nm.

### 4.12. GFP Reporter Gene Assay

HEK293T cells were transfected with pGF-AP-1-mCMV-EF1-Puro, pGF-NF-κB-mCMV-EF1-Puro, and pGF-MMP-1-mCMV-EF1-Puro vectors, using jetPEI reagent. Virus particles were collected, filtered, and used to infect HaCaT cells in the presence of polybrene. After selection with puromycin, cells were incubated in serum-free DMEM, treated with BSE, and then exposed to PM. GFP fluorescence was measured using a Cell Imaging Multi-Mode Reader (Biotek, VT, USA).

### 4.13. Kinase Assay

The kinase assay was performed according to Promega’s instructions (Madison, WI, USA). Active kinases were incubated with BSE and a substrate in kinase buffer, followed by ATP addition (150 μM for p38α and 50 μM for ERK1). Luminescence was measured using a luminescence reader (Biotek, VT, USA).

### 4.14. Pull-Down Assay

Following Kim’s method [44], Sepharose 4B beads were activated with 1 mM HCl and coupled with BSE. For the pull-down assay, HaCaT cell lysate or active p38α was incubated with BSE-Sepharose 4B beads. After washing, proteins bound to the beads were analyzed via Western blot.

### 4.15. ATP and BSE Competition Assay

Active p38α (10 ng) was incubated with BSE-Sepharose 4B beads, and ATP was added at concentrations of 10, 100, and 1000 μM. After incubation, beads were washed, and bound proteins were analyzed by Western blot.

### 4.16. Statistical Analysis

All experiments were repeated at least three times. Data were analyzed using SPSS Statistics version 21.0 (IBM, New York, NY, USA). One-way ANOVA followed by Duncan’s Multiple Range test was used to compare groups, with significance set at *p* < 0.05.

## Figures and Tables

**Table 1:** Antioxidant effects of BSE.

Method	ABTS	DPPH	Total Phenolic Content
Comparison	Vitamin C equivalents	Vitamin C equivalents	Tannic acid equivalents
Unit	mg VCEAC ^1)^/g	mg VCEAC/g	Mg TAE ^2)^/g
Value	62.86 ± 0.04 VCEAC	32.77 ± 0.28	83.68 ± 1.21

^1)^ Vitamin C equivalent Antioxidant Capacity ^2)^ Tannic acid equivalent.

## Data Availability

The data presented in this study are available on request from the corresponding author.
